# Comparison of titanium elastic intramedullary nailing versus injection of bone marrow in treatment of simple bone cysts in children: a retrospective study

**DOI:** 10.1186/s12891-016-1184-7

**Published:** 2016-08-15

**Authors:** Wenchao Li, Ruijiang Xu, Minghua Du, Hui Chen

**Affiliations:** Department of Pediatric Orthopaedic Surgery, Chinese People’s Liberation Army General Hospital, 28 Fuxing Road, Beijing, 100853 China

**Keywords:** Simple bone cyst, Elastic intramedullary nailing, Bone marrow, Long bones, Children

## Abstract

**Background:**

Simple bone cysts are common benign lytic bone lesions in children. The main goals of treatment for bone cysts are to prevent pathological fractures, support the healing process, and prevent recurrence. This retrospective study compared fixation with titanium elastic intramedullary nailing (TEN) versus aspiration and injection of autogenous bone marrow (ABM) for the treatment of simple bone cysts in children.

**Methods:**

Forty-six patients (mean follow-up, 62 months; range, 34–71 months) who presented with bone cysts (30 in the humerus, 16 in the femur) from January 2006 to December 2012 were retrospectively evaluated. Patients were treated with either TEN or ABM injection. Radiographs were evaluated according to previously established criteria. Clinical evaluations of pain, infection, additional fractures, and range of motion were performed.

**Results:**

After treatment, all patients were pain-free and had normal range of motion in adjacent joints. In the ABM group, 14 (60.9 %) bone cysts completely healed, six (26.1 %) healed with small residuals after two injections, and three (13.0 %) recurred. In the TEN group, 16 (69.6 %) bone cysts completely healed, four (17.4 %) healed with small residuals, and three (13.0 %) recurred. There were no significant differences in radiographic outcomes between groups at the final follow-up (*P* > 0.05). Three patients developed skin irritation as a result of the nail ends. Additional fractures occurred in four patients who underwent ABM injection and in two patients who underwent TEN. No significant associations were found between pathological fractures and cyst activity, location, or treatment outcomes in the patients studied.

**Conclusions:**

Both TEN and ABM injection are safe and effective treatment for bone cysts. ABM injection promotes osteogenic differentiation of bone marrow stromal cells; multiple injections can reduce the likelihood of recurrence. TEN stabilizes the affected bone and thus allows early limb mobilization. It also reduces pressure in the capsule wall by continuous decompression to promote cyst healing. ABM injections can be used to treat cyst recurrence after previous TEN, with favorable results.

## Background

Bone cysts are common benign lytic bone lesions in children. These lesions mainly occur in the proximal humerus and femur. They are usually diagnosed after pathological fracture but may be an incidental finding. Simple bone cysts are believed to be self-limiting because they disappear with skeletal maturity. The main goals of treatment are thus prevention of pathological fractures, support of the healing process, and prevention of recurrence [1, 2].

Treatments for bone cysts include curettage and bone grafting, aspiration and injection of autogenous bone marrow (ABM) or methylprednisolone, and fixation with titanium elastic intramedullary nail (TEN). Curettage and bone grafting were historically the preferred treatment for bone cysts. However, the results were disappointing because of the high rate of recurrence [3, 4]. Aggressive surgical curettage has been associated with complications including infection, coxa vara, physeal damage, limb shortening, increased blood loss, pathological fracture, and a prolonged period of postoperative immobilization [1]. Scaglietti [5] reported that local injection of methylprednisolone acetate had a favorable outcome rate of 90 % in the treatment of bone cysts. However, the recurrence rate following steroid injection is as high as 13 %, and the treatment commonly requires several injections [6]. Limb-length discrepancies have also been reported in 5 to 15 % of patients treated with steroid injection [7, 8].

Biological treatment methods such as injection of autogenous or allogeneic ABM or bone substitutes can reportedly optimize results [9]. Autogenous ABM with osteoinductive potential has been used as an alternative to curettage and bone grafting [6]. Aspiration of bone cysts is sufficient to relieve pressure in the cyst and evacuate the cyst fluid, which has high bone-resorbing activity. Bone marrow has osteogenic capacity, promoting cyst healing as well as any other material [10]. Multiple injections can achieve adequate obliteration of bone cysts in some cases [11]. However, such aspiration and percutaneous treatments do not provide early mechanical stabilization of the weakened bone [12].

TEN has been used for treatment of unicameral bone cyst associated with long bone fracture, with favorable results [13]. TEN can provide prompt stabilization of the affected bone to prevent pathological fracture and allow for early mobilization [14]. Pogorelić [15] reported that 18 bone cysts treated with TEN healed completely with no recurrence. In a study by Masquijo [16], unicameral bone cysts were treated by continuous decompression with TEN; a successful outcome was observed in 89.5 % of cases (26 total healing, 17 healing with residual radiolucent areas), and four cysts recurred.

Both ABM and fixation with TEN have been conventional treatment methods for bone cysts in our department. In the present study, we retrospectively reviewed cases of bone cyst treated with ABM or TEN. The purpose of the study was (1) to compare the clinical outcomes and healing of bone cysts after ABM versus TEN and (2) to evaluate the characteristics of ABM and TEN in the treatment of simple bone cysts in children.

## Methods

We performed a retrospective review of 46 patients with simple bone cysts treated from January 2006 to December 2012. Bone cysts were diagnosed based on their characteristic radiographic appearance and histological findings. After radiographic diagnosis, bone cysts were observed without treatment for 3 to 6 months. If bone cysts showed improvement during this time, observation continued with follow-up at 3-month intervals. If bone cysts showed progression or if pathological fracture occurred, ABM or TEN was performed. The surgeon chose the treatment method based on factors such as the location of the bone cyst and the presence of fracture. Thirty cysts occurred in the humerus and 16 in the femur. Patients with pathological fracture underwent immobilization with a brace for 3 weeks before surgical intervention. Informed consent to undergo treatment with ABM injection or TEN was obtained from all patients and their parents or guardians. Patients who had previously received any treatment other than immobilization for pathological fractures were excluded from the study, as were patients lost during follow-up. The choice of treatment was based on several factors, including advantages of the respective methods, patient preference, characteristics of the bone cysts, the necessity of eventual surgery to remove the nails with TEN, and the possible need for multiple surgeries with ABM. Patients or guardians helped to decide on the appropriate treatment before surgery. Informed consent to undergo treatment with ABM injection or TEN was obtained from all patients and their parents or guardians. TEN provides mechanical stability of pathological fractures; therefore bone cysts in the calcar femorale and those with fractures reducing the strength of cortical bone were generally treated with TEN. The flow of bone marrow can be blocked in multiseptated bone cysts, which can result in cyst recurrence with ABM treatment. TEN can break the septation in bone cysts, allowing the free flow of cyst fluid and reducing cyst pressure.

## ABM aspiration and injection technique

Surgery was performed under general anesthesia. Fluoroscopy was used intraoperatively to determine the position of bone cysts. Percutaneous bone marrow puncture was performed, and clear or straw-colored fluid was aspirated. For multilocular bone cysts, each cavity was aspirated separately. After removal of all cyst fluid, bone marrow was aspirated from the iliac crest and injected into the cyst. The volume of bone marrow aspirated from a single site was 2 to 3 ml. Most patients received 10 to 15 ml, collected from several sites on the iliac crest. If a cyst was not completely healed after 8 months, another injection was performed.

## TEN technique

TEN was performed for internal fixation. The diameter of the intramedullary nail (Synthes, Solothurn, Switzerland) ranged from 2 to 4 mm, with 0.5-mm increments between available diameters. The size and length were chosen based on the size of the bone on operative radiographs. Incisions approximately 1 cm in length were made medially and laterally in the distal limb. The medullary canal was opened an adequate distance from the growth plate. The nail was gradually introduced into the medullary canal and through the bone cyst under fluoroscopic guidance. Nail stability was verified by rotating the limb under fluoroscopy. The nail end was trimmed to avoid skin irritation. If necessary, a second nail was placed using the same method. Patients gradually returned to their normal activities 24 h after surgery. Full activity, including weight bearing, was resumed 4 weeks postoperatively.

Clinical and radiographic assessments were performed 6 and 12 weeks after surgery and again after 6 to 24 months. Clinical evaluations for pain, infection, additional fractures, and range of motion were also performed. The radiographic criteria described by Capanna et al. [17] were used to classify the healing of bone cysts: Grade I, completely healed; Grade II, healed with small residuals; Grade III, recurrence of an initially healed cyst with increasing signs of radiolucency and cortical thinning; and Grade IV, no response after surgery. Grades I and II represent successful treatment, while Grades III and IV represent treatment failure. The radiographic outcomes were evaluated by radiologists and orthopedic surgeons. If disagreement occurred regarding a radiograph, an extra evaluation was performed by double-blinded radiologists and orthopedic surgeons.

Pearson’s chi-squared test was used to compare general data and the condition of bone cyst healing between the two treatment groups. Fisher’s exact test was used for theoretical samples of less than five. P values <0.05 were considered statistically significant. Kappa values were used to determine intraobserver and interobserver repeatability and reliability.

## Results

Sixteen bone cysts were located in the humerus and seven in the femur in the ABM group, whereas 14 were located in the humerus and nine in the femur in the TEN group (*P* > 0.05). The mean age at diagnosis was 7.9 ± 3.3 years in the ABM group and 8.3 ± 3.1 years in the TEN group (*P* > 0.05). The mean age at surgery was 8.8 ± 2.9 years and 9.1 ± 3.2 years, respectively (*P* > 0.05). There were 14 male and nine female patients in the ABM group and 15 male and eight female patients in the TEN group (*P* > 0.05). The mean follow-up period was 62 months (range, 34–71 months). There was no significant difference in cyst location, sex, age at diagnosis, or age at first surgery between the two groups (*P* > 0.05). Eight patients had active cysts and 15 had latent cysts in the ABM group, while 11 patients had active cysts and 12 had latent cysts in the TEN group (*P* > 0.05). There was no significant difference in cyst activity, location, or presence of fracture between the two groups (*P* > 0.05). The interobserver reliability ranged from 73.3 to 86.7 % (Kappa coefficient, 0.761–0.847). Intraobserver repeatability ranged from 83.3 to 93.3 % (Kappa coefficient, 0.821–0.925) (*P* < 0.01).

In the ABM group, 14 (60.9 %) patients had pathological fractures, five (21.8 %) had pain or loss of function with no radiographic evidence of fracture, and four (17.3 %) had cysts diagnosed as an incidental radiographic finding. In the TEN group, 15 (65.2 %) patients had pathological fractures, five (21.8 %) had pain or loss of function without fracture, and three (13.0 %) had cysts diagnosed as an incidental finding. There was no significant difference in the reason for detection of bone cysts between the two groups (*P* > 0.05). None of the fractures involved a growth plate (Table 1).

**Table 1:** Demographic data and general data for fixation of flexible intramedullary nailing and aspiration of injection of bone marrow

Parameter	Aspiration of injection of bone marrow	Fixation of flexible intramedullary nailing	*P* value
Number of patients	23	23	-
Femur	7 (30.4 %)	9 (39.1 %)	0.75
Humeral	16 (69.6 %)	14 (60.9 %)	
Age at diagnosis^a^	7.9 ± 3.3	8.3 ± 3.1	0.67
Age at first surgery^a^	8.8 ± 2.9	9.1 ± 3.2	0.74
Female	9 (39.1 %)	8 (37.8 %)	1.00
Male	14 (60.9 %)	15 (65.2 %)	
Cyst activity (cm)^a^ (distance from physis)^a^	2.1 ± 2.2	1.9 ± 2.1	0.75
Active	8 (34.8 %)	11 (47.8 %)	0.55
Latent	15 (65.2 %)	12 (52.2 %)	
Unilocular	10 (43.5 %)	9 (39.1 %)	1.00
Multilocular	13 (56.5 %)	14 (60.9 %)	
Fractures	14 (60.9 %)	15 (65.2 %)	0.84
Pain or inability without fracture	5 (21.8 %)	5 (21.8 %)	
Incidental findings	4 (17.3 %)	3 (13.0 %)	

^a^The values in the groups columns are given as the mean and standard deviation

**Fig. 1:**
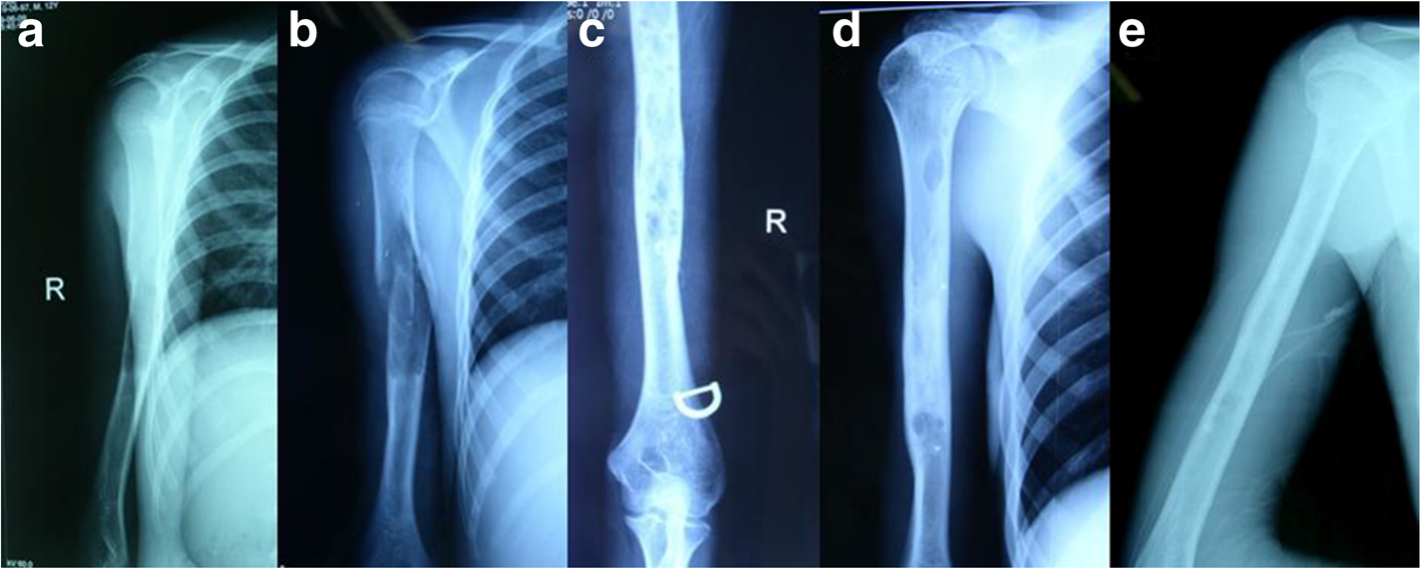
Radiographs of a 7-year-old boy evaluated for arm pain and loss of function. **a** A bone cyst is present in the proximal humerus with deviation of the axis. **b** A significantly displaced pathological fracture occurred after a sudden fall. **c** The lesion shows signs of healing after aspiration and injection of bone marrow. **d** The bone cyst is healed 18 months postoperatively. **e** The radiograph shows the bone cyst healed at 68-month follow-up

**Fig. 2:**
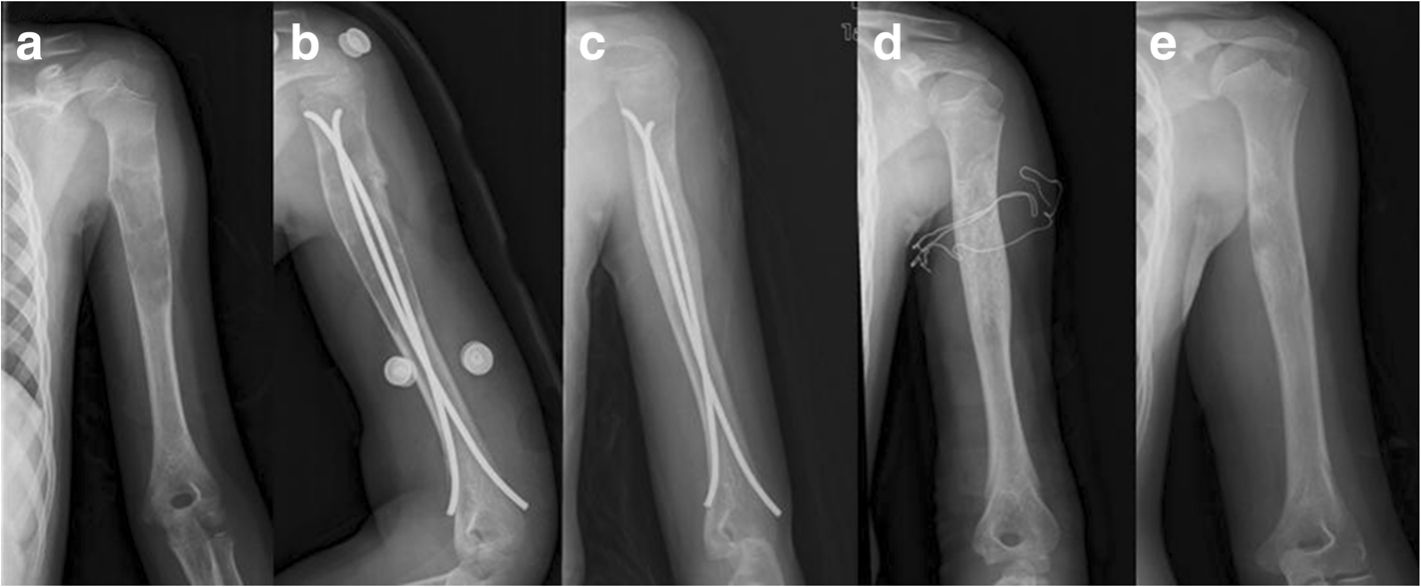
Radiographs of an 8-year-old girl who presented with pathological fracture of the right humerus. **a** Anteroposterior radiograph at presentation. A bone cyst with pathological fracture is located in the proximal metaphysis and diaphysis of the humerus. **b** Fixation with flexible intramedullary nails was performed. **c** Three months after surgery the lesion is significantly smaller and shows sings of healing. **d** At 28 months the bone cyst has resolved, and the intramedullary nails are removed. **e** At 61-month follow-up the bone cyst is completely healed without refracture or deviation of the axis

**Fig. 3:**
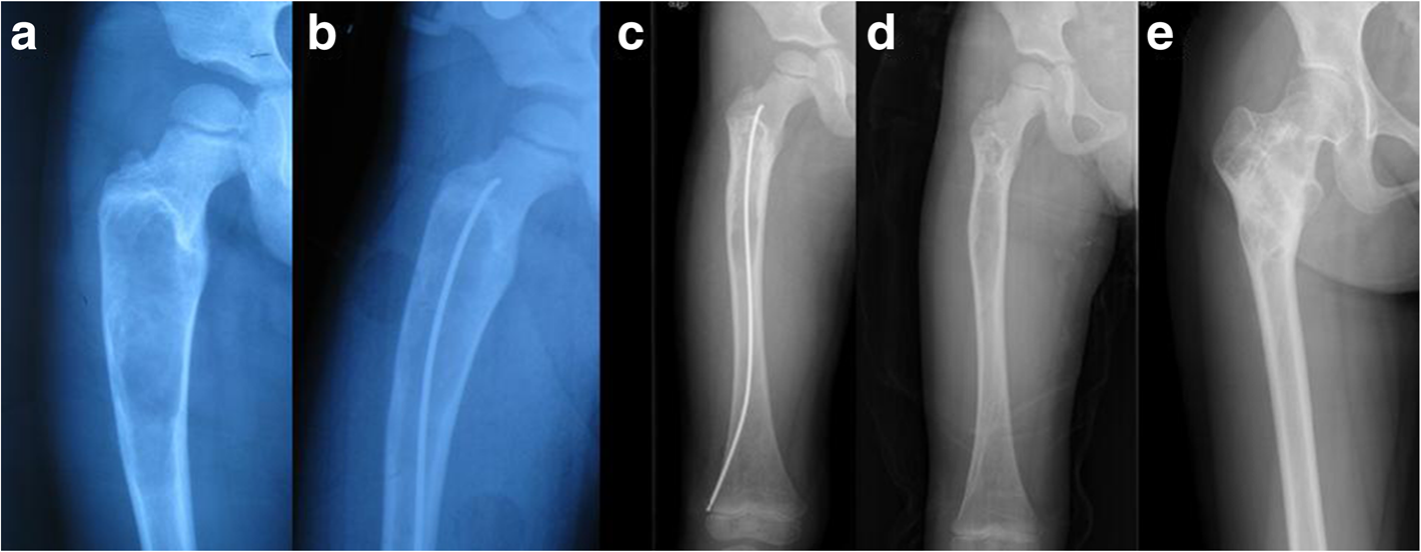
Radiographs of a 5-year-old boy who presented with leg pain and loss of function. **a** A bone cyst is present in the proximal metaphysis of the femur without pathological fracture. **b** Flexible intramedullary nailing was used to fix and decompress the bone cyst. **c** The lesion shows signs of healing 6 months after surgery. **d** At 26 months the bone cyst is healed and the nail is removed. **e** The lesion is completely healed at 59-month follow-up

According to the criteria described by Capanna et al. [17], 14 (60.9 %) bone cysts in the ABM group achieved Grade I (complete healing) and six (26.1 %) achieved Grade II (healing with small residuals) at 18 months after two injections (Fig. 1). Three (13.0 %) cysts improved initially and subsequently recurred. In these cases, two ABM injections were performed and the bone cysts were healed with residuals after 6 months. In the TEN group, 16 (69.6 %) bone cysts achieved Grade I and four (17.4 %) cysts achieved Grade II after 18 months. The intramedullary nails were removed after 18 months (Fig. 2). Radiographically, two (8.7 %) limbs showed slight deformities in the sagittal and frontal planes. Three (13.0 %) cysts gradually improved and then recurred after 12 months. In cases of recurrence, ABM injection was performed and the bone cysts were healed with small residuals after 8 months. No patient in either group had Grade IV cysts. There was no significant difference in the outcomes between the two groups at the final follow-up, according to the criteria of Capanna et al. [17] (*P* > 0.05) (Table 2, Fig. 3).

**Table 2:** The treatment outcomes of flexible intramedullary nailing and injection of bone marrow

Parameter	Aspiration of injection of bone marrow	Fixation of flexible intramedullary nailing	*P* value
Complications			
Skin irritation	0	3 (13.0 %)	-
Additional fracture	4 (17.3 %)	1 (4.3 %)	-
Skin infection	3 (13.0 %)	1 (4.3 %)	-
The first outcome by criteria of Capanna et al^a^			
Grade I	14 (60.9 %)	16 (69.6 %)	0.77
Grade II	6 (26.1 %)	4 (17.4 %)	
Grade III	3 (13.0 %)	3 (13.0 %)	
Grade IV	0	0	
One injection	7	3^b^	-
Twice injection	12	0	-
Four injection	3	0	-

^a^ The first outcome was the result by two injections of bone marrow or fixation of intramedullary nailing after 18 month postoperatively. ^b^ The three patients in Grade III were preformed with injection of bone marrow as a result of healing of bone cysts with small residuals

On clinical evaluation, all patients were pain-free and had normal range of motion in the joints after surgery. Skin irritation resulting from nail ends developed in three (13.0 %) patients in the TEN group; irritation gradually resolved after nail removal. Pathological fractures developed in four (17.3 %) patients in the ABM group and in one (4.3 %) in the TEN group. In these cases a plaster cast was applied for 4 weeks, and the bone cysts healed to Grade II. Three (13.0 %) patients in the ABM group and one (4.3 %) in the TEN group developed skin infections. These infections healed well with antibiotic treatment.

Our subsequent analysis explored the relationships between the various factors associated with pathological fractures in all patients (Table 3). Eleven of 16 femoral cysts and 18 of 30 humeral cysts were associated with pathological fracture (*P* < 0.05). Among all 29 patients with fracture, 21 cysts healed to Grade I, six to Grade II, and two to Grade III. Among the 17 patients without fracture, nine cysts healed to Grade I, four to Grade II, and four to Grade III. There was no significant association between pathological fracture and the form of the bone cyst (unilocular versus multilocular) (*P* > 0.05). There were no significant associations between pathological fracture and cyst activity or outcome according to the criteria of Capanna et al. [17] (*P* > 0.05).

**Table 3:** The relative factors associated with pathological fracture

Parameter	Fracture	No fracture	*P* value
Number	29	17	-
Femur	11 (37.9 %)	5 (29.4 %)	0.79
Humeral	18 (62.1 %)	12 (70.6 %)	
Cyst activity^a^	2.0 ± 2.2	2.5 ± 2.7	0.49
Unilocular	12 (41.4 %)	7 (41.2 %)	1.00
Multilocular	17 (58.6 %)	10 (51.5 %)	
The first outcome by criteria of Capanna et al^b^			
Grade I	21	9	0.23
Grade II	6	4	
Grade III	2	4	

^a^The values in the groups columns are given as the mean and standard deviation

^b^The first outcome was the result by two injections of bone marrow or fixation of intramedullary nailing after 18 month postoperatively

## Discussion

Simple bone cysts are common bone lesions in children first described by Virchow two centuries ago. The etiology of bone cysts is uncertain; possible etiologies include localized failure of ossification, entrapped intramedullary synovial tissue, or restricted blood supply resulting from venous blockage. Simple bone cysts are prone to pathological fracture resulting from minor injury, particularly enormous bone cysts of the calcar femorale. Bone cysts are usually discovered because of pathological fracture but can be incidental findings. Active bone cysts in the humerus or femur are gradually progressive. Though simple bone cysts are often asymptomatic and tend to disappear with skeletal maturity, repeated pathological fractures and growth arrest can occur before spontaneous resolution. Therefore, the goal of treatment is to prevent pathological fractures and the resulting skeletal deformities during growth [18]. The treatment methods for bone cysts include curettage and grafting, aspiration and injection, damage to the cyst wall and lining, cyst decompression, mechanical support, or a combination of these strategies. At our institution, a percutaneous technique is used instead of open operations such as curettage and grafting. The percutaneous technique is safe and effective; ABM, methylprednisolone acetate, cortisone, demineralized bone matrix, calcium-phosphate bone cement, and intramedullary nails can be applied to bone cysts with various results [1, 6, 18, 19]. In this study, we retrospectively reviewed patients treated with ABM injection or TEN to evaluate clinical outcomes and radiographic findings. There was no significant differences in outcomes between the ABM and TEN groups at the final follow-up, according to the criteria of Capanna et al. [17] (*P* > 0.05). However, TEN and ABM injection have different characteristics during the treatment process.

Lokiec et al. [20] first introduced ABM grafting for bone cyst treatment with successful results. Bone marrow injection provides osteoprogenitor cells, stimulates new bone formation, and promotes healing of bone cysts because of its osteoinductive properties. Clinical studies have suggested that aspirated bone marrow promotes bone cyst resolution [20]. Osteoblast progenitor cells and bone marrow stem cells have the potential to differentiate into osteoblasts. The optimum volume of bone marrow to inject is defined by the osteoblast progenitor cells. The number of cells increases as the aspiration volume increases [21]. However, the number of cells at the site of the iliac bone is limited. When the volume of aspirated bone marrow increases to more than 2 to 3 ml, the concentration of osteoblast progenitor cells is reduced as peripheral blood is mixed with the bone marrow. To maximize the number of available cells, we recommend that total volume per site in the iliac bone should be limited to 2 to 3 ml, similar to the recommendation made in another study [9].

A second ABM injection was performed in this study in patients with incomplete healing at 8 months postoperatively. Two injections can promote osteogenic differentiation and reduce the cyst recurrence rate. Fourteen (60.9 %) bone cysts completely healed to Grade I, and six (26.1 %) healed to Grade II. In a study of ABM treatment in patients with bone cysts, Docquier et al. [22] reported that 13 (76 %) cases exhibited slow regression and progressive healing, two exhibited no response, and two developed recurrence. Bone cysts in three (13.6 %) patients gradually diminished to about one-third of their original size at 12 months with two injections. Another two injections were performed for partially healed cysts with residuals. Zamzam et al. [9] reported that repeated injections of ABM are necessary to reduce of the recurrence of bone cysts. Other authors have also recommended multiple injections of ABM in the treatment of bone cysts [2]. Connolly et al. [23] reported that there is no harm in multiple injections and that they may be beneficial in many cases.

According to previous studies, TEN has rapidly been gaining popularity as a treatment for simple bone cysts in long bones. Pogorelić et al. [15] reported that 18 bone cysts treated with TEN were healed completely at 3 months with no cyst recurrence. De Sanctis et al. [24] reported that 31 (65.9 %) of 47 cysts treated with TEN completely healed and that 16 (34.1 %) healed with residual radiolucency. Glanzmann et al. [25] performed TEN to treat 20 patients; 16 cysts healed completely and four healed with residual areas without recurrence. Kanellopoulos et al. [1] reported that seven of nine cysts consolidated completely (Neer stage I) and that two of nine cysts consolidated partially (Neer stage II).

The choice of treatment between ABM and TEN in this study was based on several factors, including advantages of the respective methods, patient preferences, characteristics of the bone cyst, the necessity of surgery for nail removal in TEN, and the need for multiple procedures in ABM. Patients or guardians chose treatment methods before surgery. There were no significant differences in cyst location, sex, age at diagnosis, or age at first surgery between the two treatment groups (*P* > 0.05). Likewise, there were no significant differences in cyst activity, location, or presence of fractures between the two groups (*P* > 0.05). We plan to perform a prospective study on the treatment of bone cysts in the future.

The presence of bone cysts affect bone strength, and pathological fractures are a common reason for hospitalization. Bone cysts in the proximal femur are generally located in the calcar femorale, which plays a significant role in the strength of the intertrochanteric region of the femur and stability of intertrochanteric fractures. Lokiec et al. [20] reported that percutaneous injection of ABM could weaken the consolidation of bone cysts. Additionally, aspiration of cyst fluid with percutaneous injection does not initially enhance the mechanical stability of cysts. Intramedullary nails provide mechanical stabilization of pathological fractures in the femur and humerus. Nail fixation is invasive surgery, but does not destroy the structure of the bone cyst. TEN has two main benefits: continuous cyst decompression and early immediate stabilization of the involved bone, which permits early mobilization and a return to the normal activities of pre-teen patients [1]. Journeau et al. [12] reported that TEN should be preferred for weight-bearing segments, regardless of whether the bone cyst is associated with fracture. The authors recommended that patients undergoing ABM injection should not be allowed to perform strenuous exercise for 1 month after the procedure. Bone cysts in the proximal femur, especially those in the calcar femorale, should be treated with fixation by TEN.

Decompression of bone cysts is performed to treat venous obstruction that leads to intramedullary accumulation and later cavity formation [26]. Percutaneous drilling of multiple sites with Kirschner wires and prolonged decompression of bone cysts with cannulated screws or intramedullary nails left in place have been advocated for the treatment of bone cysts. Drilling leads to decompression and decreases the pressure within the bone cyst [27, 28]. TEN decompresses bone cysts and breaks down the capsule wall to allow the capsule fluid to flow into the medullary cavity. Decompression of bone cysts reduces the pressure on the cortex and promotes regeneration of newly formed bone [3]. We repeatedly rotated the nail in the bone cyst to break down the structure of the capsule wall, which reduces the production of capsule fluid by the cyst and decompresses the bone cyst. Some authors have recommended performing multiple perforations of the cyst wall, breaking the intralesional septa, and/or curetting the cyst membrane [19, 25]. Glanzmann et al. [25] reported that it was unclear whether insertion of nails and consecutive decompression of bone cysts can lead to migration of pathological tissue, provoking recurrence. However, that study compared intramedullary nailing with steroid injection for bone cysts and found no difference in healing times and fewer complications in patients who underwent nailing [12]. In a study by Masquijo [16], unicameral bone cysts were treated with continuous decompression with intramedullary nailing. In that study, successful results were observed in 89.5 % of cases (26 total healing, 17 healing with residual radiolucent areas); four cysts recurred.

In the TEN group in the present study, bone cysts in 16 (69.6 %) patients were completely healed within 12 months. Four (17.4 %) bone cysts had healed with residuals at the last follow-up. Glanzmann et al. [25] reported that 16 of 22 cysts treated with TEN healed completely and four healed to Grade II, with a minimal complication rate. TEN should be preferred for treatment of bone cysts, based on a success rate of 94 % in 32 unicameral bone cysts. The bone cysts in three patients gradually diminished initially and recurred after 6 months. After injection of bone marrow, these bone cysts healed with small residuals after 12 months. The combined biological and mechanical treatment of simple bone cysts has also been described in recent studies. Injection of ABM and demineralized bone matrix in addition to TEN has also been used for stabilization of bone cysts [1]. Rappet al. [29] reported that the combination of TEN, artificial bone substitute, and autologous platelet-rich plasma enhanced the treatment of bone cysts in children, with no resulting complications.

## Conclusion

Both fixation by TEN and ABM injection are safe and effective methods for treatment of bone cysts. After treatment with ABM or TEN, the expected natural history is that the cyst gradually shrinks and disappears after skeletal maturation. Fixation with TEN and limiting activity may improve the healing of bone cysts and prevent pathological fracture in children. ABM injection induces osteogenic differentiation of bone marrow stromal cells; multiple injections can reduce the likelihood of cyst recurrence. TEN provides stabilization of the affected bone and thus allows early limb mobilization. TEN also allows continuous decompression, which reduces the pressure on the capsule wall and promotes bone cyst healing. ABM injection can be used to treat recurrent cysts after previous TEN, with favorable results.

## Abbreviations

ABM, injection of autogenous bone marrow; TEN, titanium elastic intramedullary nailing

## References

[1] Kanellopoulos AD, Mavrogenis AF, Papagelopoulos PJ, Soucacos PN (2007). Elastic intramedullary nailing and DBM-bone marrow injection for the treatment of simple bone cysts. World J Surg Oncol.

[2] Kadhim M, Sethi S, Thacker MM (2015). Unicameral bone cysts in the humerus: treatment outcomes. J Pediatr Orthop.

[3] Dormans JP, Sankar WN, Moroz L, Erol B (2005). Percutaneous intramedullary decompression, curettage, and grafting with medical-grade calcium sulfate pellets for unicameral bone cysts in children: a new minimally invasive technique. J Pediatr Orthop.

[4] Flont P, Malecki K, Niewola A, Lipczyk Z, Niedzielski K (2015). Predictive characteristic of simple bone cyst treated with curettage and bone grafting. BMC Musculoskelet Disord.

[5] Sakamoto A, Matsuda S, Yoshida T, Iwamoto Y (2010). Clinical outcome following surgical intervention for a solitary bone cyst: emphasis on treatment by curettage and steroid injection. J Orthop Sci.

[6] Canavese F, Wright JG, Cole WG, Hopyan S (2011). Unicameral bone cysts: comparison of percutaneous curettage, steroid, and autologous bone marrow injections. J Pediatr Orthop.

[7] Chang CH, Stanton RP, Glutting J (2002). Unicameral bone cysts treated by injection of bone marrow or methylprednisolone. J Bone Joint Surg (Br).

[8] Tsuchiya H, Abdel-Wanis ME, Uehara K, Tomita K, Takagi Y, Yasutake H (2002). Cannulation of simple bone cysts. J Bone Joint Surg (Br).

[9] Zamzam MM, Abak AA, Bakarman KA, Al-Jassir FF, Khoshhal KI, Zamzami MM (2009). Efficacy of aspiration and autogenous bone marrow injection in the treatment of simple bone cysts. Int Orthop.

[10] Di Bella C, Dozza B, Frisoni T, Cevolani L, Donati D (2010). Injection of demineralized bone matrix with bone marrow concentrate improves healing in unicameral bone cyst. Clin Orthop Relat Res.

[11] Saraph V (2004). Treatment of simple bone cyst using bone marrow injection. J Pediatr Orthop.

[12] Cha SM, Shin HD, Kim KC, Kang DH (2013). Flexible intramedullary nailing in simple bone cysts of the proximal humerus: prospective study for high-risk cases of pathologic fracture. J Pediatr Orthop B.

[13] de Sanctis N, Andreacchio A (2006). Elastic stable intramedullary nailing is the best treatment of unicameral bone cysts of the long bones in children?: Prospective long-term follow-up study. J Pediatr Orthop.

[14] Rapp M, Svoboda D, Wessel LM, Kaiser MM. Elastic Stable Intramedullary Nailing (ESIN), Orthoss(R) and Gravitational Platelet Separation--System (GPS(R)): an effective method of treatment for pathologic fractures of bone cysts in children. BMC Musculoskelet Disord. 12:45. doi: 10.1186/1471-2474-12-45 10.1186/1471-2474-12-45 PMC3046000 21314981

[15] Pogorelic Z, Furlan D, Biocic M, Mestrovic J, Juric I, Todoric D (2010). Titanium intramedullary nailing for treatment of simple bone cysts of the long bones in children. Scott Med J.

[16] Masquijo JJ, Baroni E, Miscione H (2008). Continuous decompression with intramedullary nailing for the treatment of unicameral bone cysts. J Child Orthop.

[17] Capanna R, Albisinni U, Caroli GC, Campanacci M (1984). Contrast examination as a prognostic factor in the treatment of solitary bone cyst by cortisone injection. Skeletal Radiol.

[18] Cho HS, Seo SH, Park SH, Park JH, Shin DS, Park IH (2012). Minimal invasive surgery for unicameral bone cyst using demineralized bone matrix: a case series. BMC Musculoskelet Disord.

[19] Lakhwani OP (2013). Percutaneous method of management of simple bone cyst. Case Rep Orthop.

[20] Lokiec F, Wientroub S (1998). Simple bone cyst: etiology, classification, pathology, and treatment modalities. J Pediatr Orthop B.

[21] Hyer CF, Berlet GC, Bussewitz BW, Hankins T, Ziegler HL, Philbin TM (2013). Quantitative assessment of the yield of osteoblastic connective tissue progenitors in bone marrow aspirate from the iliac crest, tibia, and calcaneus. J Bone Joint Surg Am.

[22] Docquier PL, Delloye C (2003). Treatment of simple bone cysts with aspiration and a single bone marrow injection. J Pediatr Orthop.

[23] Connolly JF (1998). Clinical use of marrow osteoprogenitor cells to stimulate osteogenesis. Clin Orthop Relat Res.

[24] de Sanctis N, Andreacchio A (2006). Elastic stable intramedullary nailing is the best treatment of unicameral bone cysts of the long bones in children? Prospective long-term follow-up study. J Pediatr Orthop.

[25] Glanzmann MC, Campos L (2007). Flexible intramedullary nailing for unicameral cysts in children's long bones : Level of evidence: lV, case series. J Child Orthop.

[26] Spinner RJ, Wang H, Carmichael SW, Amrami KK, Scheithauer BW (2007). Epineurial compartments and their role in intraneural ganglion cyst propagation: an experimental study. Clin Anat.

[27] Lakhwani OP (2013). Percutaneous method of management of simple bone cyst. Case Rep Orthop.

[28] Shirai T, Tsuchiya H, Terauchi R, Tsuchida S, Mizoshiri N, Ikoma K (2015). Treatment of a simple bone cyst using a cannulated hydroxyapatite pin. Medicine (Baltimore).

[29] Rapp M, Svoboda D, Wessel LM, Kaiser MM (2011). Elastic Stable Intramedullary Nailing (ESIN), Orthoss(R) and Gravitational Platelet Separation--System (GPS(R)): an effective method of treatment for pathologic fractures of bone cysts in children. BMC Musculoskelet Disord.

